# Successful brentuximab vedotin monotherapy against late relapse of classical Hodgkin lymphoma 6 years after first remission

**DOI:** 10.1002/ccr3.2688

**Published:** 2020-01-31

**Authors:** Kana Nagashima, Shohei Kikuchi, Satoshi Iyama, Chisa Fujita, Akari Goto, Hiroto Horiguchi, Masayoshi Kobune

**Affiliations:** ^1^ Department of Hematology Sapporo Medical University School of Medicine Sapporo Japan

**Keywords:** brentuximab vedotin, classical Hodgkin lymphoma, late‐relapse

## Abstract

Brentuximab vedotin monotherapy for late‐relapse CHL is a promising therapeutic with sustained CR benefit and avoiding potential toxicities caused by aPBSCT/HDT.

## Graphical abstract

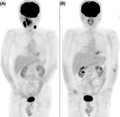

## 1 INTRODUCTION

With classical Hodgkin lymphoma (CHL), a complete response (CR) and long‐term progression‐free survival are expected in most cases after initial treatment, such as with doxorubicin, bleomycin, vinblastine, and dacarbazine (ABVD). However, about 10%‐30% of patients with advanced CHL relapse within 5 years.^1^, ^2^ The goal of treatment against relapsed CHL is to achieve a CR following long‐term disease control. In most cases, high‐dose chemotherapy with autologous hematopoietic stem cell transplantation rescue (aPBSCT/HDT) is selected with an expected sustained CR benefit compared to conventional chemotherapy alone.^3^ However, aPBSCT/HDT has the potential risk of late toxicity, including nonrelapse mortality, caused by a secondary malignancy such as myelodysplastic syndrome and/or acute myeloid leukemia, cardiac toxicity, and pulmonary complications.^4^, ^5^

A small group of CHL cases have been reported to relapse late after 5 years of a CR. A late‐relapse group showing more than 5 years of a CR was also reported to have a better prognosis than an early‐relapse group.^6^ In the late‐relapse group, a treatment option without aPBSCT/HDT would be reasonable, aiming for a sustained CR benefit and limiting toxicity and complications, including late nonrelapse mortality. However, an optimal treatment strategy against late‐relapse CHL has not yet been established.

In this study, we reported a successful case of brentuximab vedotin (BV) monotherapy as a reinduction therapy against late‐relapse CHL, 6 years after an initial diagnosis.

## 2 CASE REPORT

**Figure 1:**
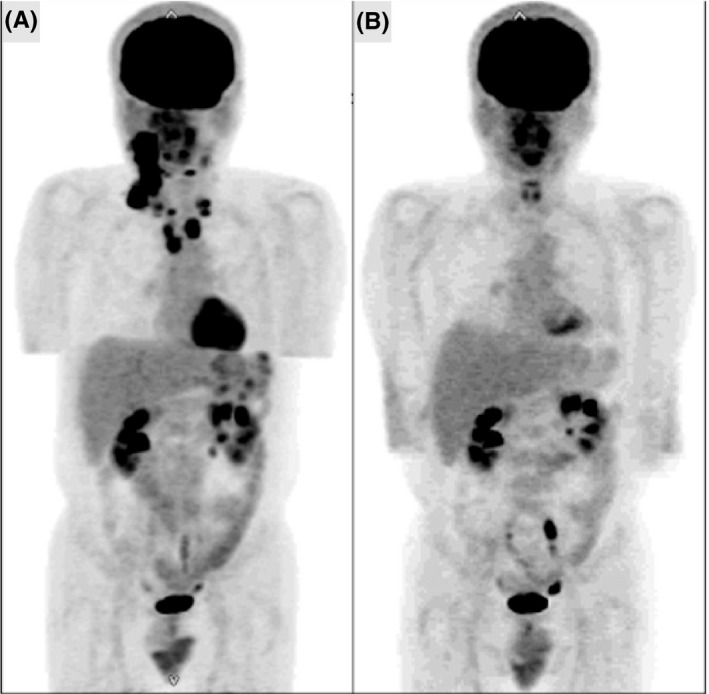
Intense uptake of 18‐fluorodeoxyglucose of the bilateral cervical and mediastinal lymphadenopathy and spleen were observed at initial diagnosis (A) and disappeared after six cycles of ABVD therapy (B) in the maximum intensity projection image (MIP) of FDG‐PET study

**Figure 2:**
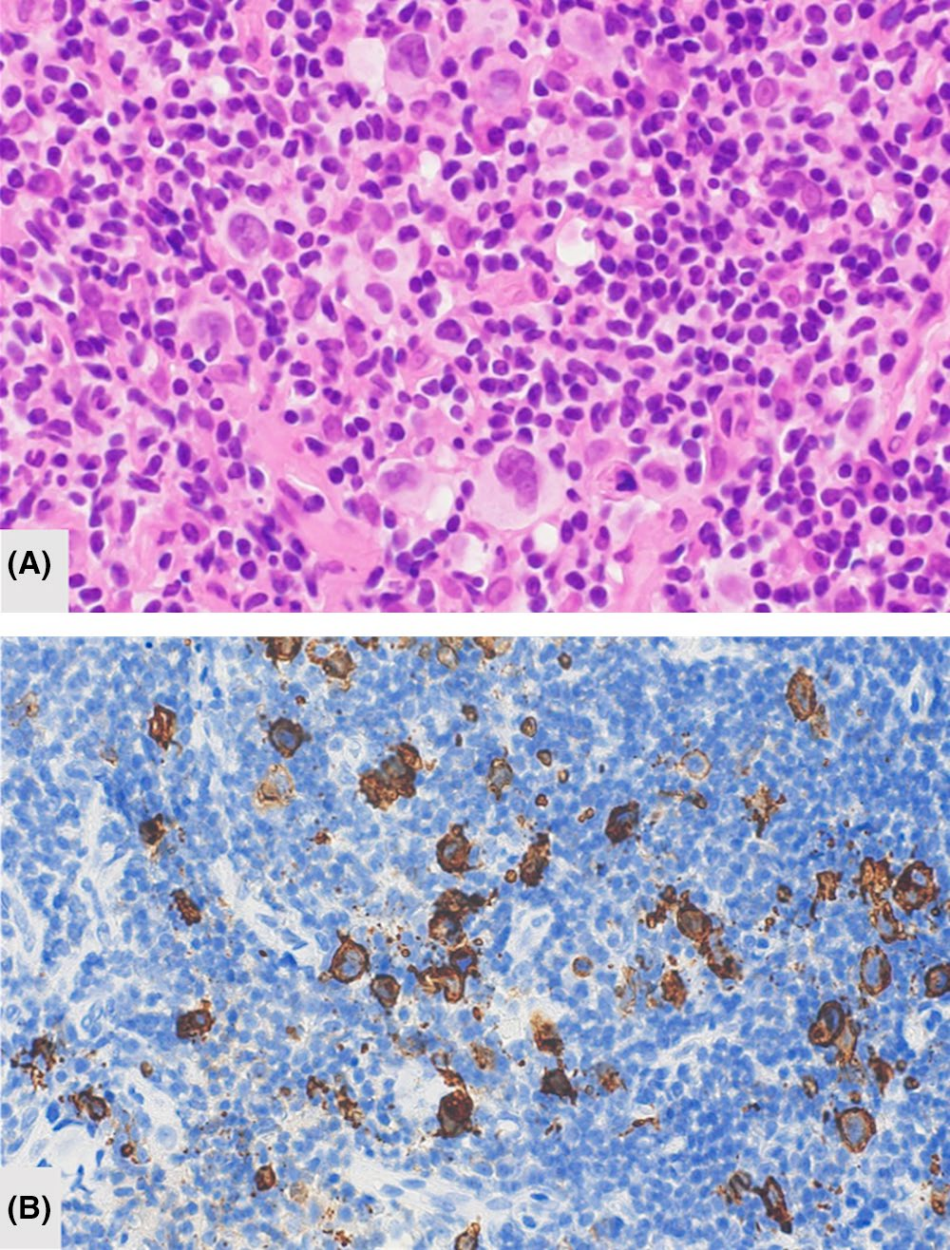
Hodgkin/Reed‐Sternberg cells are observed at hematoxylin and eosin staining (A) and stained with anti‐CD30 antibody (B) in specimen of right tonsillar biopsy at first relapse. (Original magnification ×400)

**Figure 3:**
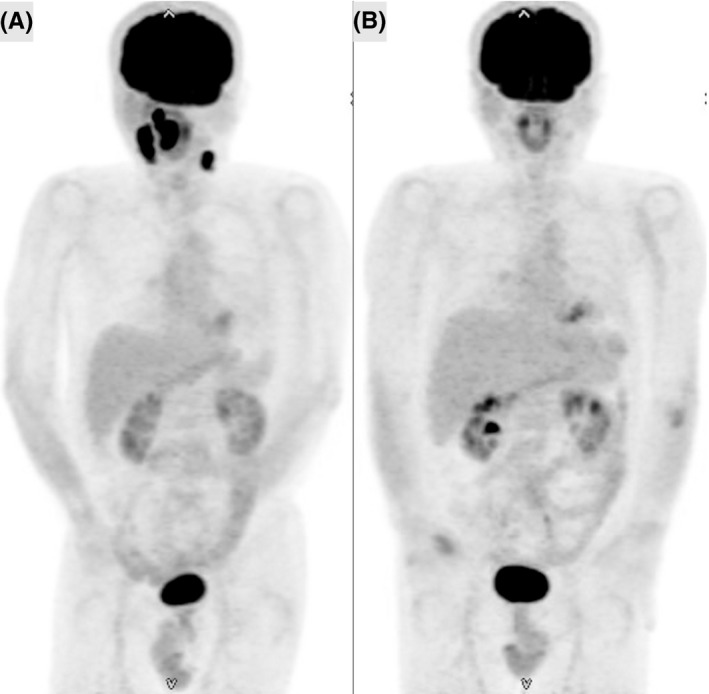
Intense uptake of 18‐fluorodeoxyglucose of the right tonsil and bilateral cervical lymphadenopathy was observed at first relapse (A) and disappeared at completion of four cycles of BV therapy (B) in the MIP image

A 52‐year‐old male patient was diagnosed with advanced CHL and had been previously successfully treated with six cycles of ABVD (Figure 1A and 1). A CR had been maintained for 6 years and recorded by medical follow‐up, including annual imaging inspections. Six years after an initial diagnosis, the patient complained of right tonsillar swelling and a subsequent physical examination revealed bilateral cervical lymph node enlargement. Relapsed CHL was histologically diagnosed by a right tonsillar biopsy (Figure 2). 18‐Fluorodeoxyglucose positron emission tomography (18^F^ FDG–PET) and contrast‐enhanced computer tomography (CT) revealed lymph node involvement of the bilateral neck region (Figure 3A). Clinical staging was restaged with IIA according to the Ann‐Arbor staging system.^7^ Because of the late‐relapse and localized involvement, reinduction therapy with BV monotherapy (1.8 mg/m^2^ q3w) was administered. We reserved aPBSCT/HDT for any possible second relapse. After one cycle of BV administration, the right tonsillar swelling and cervical lymph node enlargement physically improved and a marked adverse event was not observed. After four cycles of BV treatment, no FDG uptake was observed on the involved lesion (Figure 3B). Radiation therapy was subsequently carried out, and a CR was confirmed by 18^F^ FDG–PET performed three months after the cessation of treatment. A CR was maintained for a year and a half after BV treatment.

## 3 DISCUSSION

In CHL, a small number of patients relapse very late after initial therapy. Brockelmann reported 141 late‐relapse cases, more than 5 years after initial diagnosis, among 6840 patients, with a 10.3 years median observation period. A late‐relapse group had a better prognosis on survival than an early‐relapse group within 5 years.^6^ Early‐relapse, defined as <1 year of a CR, is considered as a poor prognostic factor as well as refractory, B symptoms, extranodal involvement^8^, ^9^ and as a good indication for aPBSCT/HDT. Radman reported that in long‐term results of conventional chemotherapy alone, a group showing a CR for more than 1 year showed better overall survival and relapse‐free survival than a group showing a CR for <1 year (10‐year overall survival of 37% vs 20% and relapse‐free survival of 40% vs 18%, respectively; *P* < .01 and <.01, respectively).^10^ Yuen also reported that in cases with a CR of more than 1 year, aPBSCT/HDT did not have significant superiority in terms of overall and event‐free survival to that of conventional chemotherapy alone.^11^ Taken together, in late‐relapse CHL, defined as a CR of at least more than 5 years, aPBSCT/HDT gives no guarantee of a survival benefit. In such groups, treatment options without aPBSCT/HDT could be optimized, aiming for a sustained CR benefit and avoiding toxicity and complications.

BV, an anti‐CD30 antibody complexed with monomethyl auristatin E, has shown a clinical effect with a 33% CR rate and a 41% overall survival rate at 5 years as salvage chemotherapy against relapsed/refractory CHL after aPBSCT/HDT.^12^ In patients who attained a CR, 38% have maintained this response for more than 5 years, highlighting the curable capability of BV monotherapy against BV‐sensitive relapsed CHL. As an induction therapy, BV combined with doxorubicin, vinblastine, and dacarbazine showed superior efficacy in the progression to ABVD against advanced CHL.^13^ BV monotherapy as a reinduction therapy is promising against late‐relapsed CHL because of not only a large clinical effect with a curative potential, but also because of the capability to prevent potential toxicity and complications of salvage chemotherapy following aPBSCT/HDT. Optimal cycles of BV monotherapy for induction or reinduction therapies are unknown. Furthermore, the additional benefit of radiation therapy after BV treatment is also unknown. In this case, after referring to the Japanese guideline recommendations for first‐line therapy for a limited stage of CHL,^14^ we chose four cycles of BV treatment following involved field radiation therapy.

In conclusion, BV monotherapy as a reinduction therapy against late‐relapse CHL is a promising therapeutic candidate. However, to elucidate any long‐term clinical effects and an optimal treatment strategy for BV monotherapy against CHL, a clinical study is needed.

## CONFLICT OF INTEREST

The authors have no conflict of interest.

## AUTHOR CONTRIBUTIONS

KN and SK: collected data and drafted the manuscript. SK, SI, CF, and AG were hematologists providing chemotherapy. All authors reviewed the manuscript. SI and MK: supervised study.
